# Serum PM20D1 levels are associated with nutritional status and inflammatory factors in gastric cancer patients undergoing early enteral nutrition

**DOI:** 10.1515/med-2024-1111

**Published:** 2025-02-07

**Authors:** Zhengyu Jin, Yuning Chen

**Affiliations:** Department of General Surgery, Suzhou Dushu Lake Hospital, Suzhou, 215000, Jiangsu, P. R. China; Department of Endocrinology and Metabolism, The Third Affiliated Hospital of Soochow University, No. 185, Juqian Street, Tianning District, Changzhou, 213000, Jiangsu, P. R. China

**Keywords:** PM20D1, gastric cancer, enteral nutrition, inflammatory factors, prognosis

## Abstract

**Background and objective:**

Early nutritional support holds paramount importance for postoperative gastric cancer (GC) patients. Peptidase M20 domain containing 1 (PM20D1) is a secretory enzyme associated with glucose and lipid metabolism. However, there is a dearth of clinical studies delving into the connection between PM20D1, lipid metabolism, and inflammatory factors in GC patients who have received enteral nutrition (EN). This research aimed to investigate the serum levels of PM20D1 in GC patients following early EN and their potential associations with lipid metabolism, nutritional markers, and inflammatory factors.

**Methods:**

This prospective observational study enrolled 180 GC patients between May 2020 and July 2022. On the first postoperative day, all patients received EN support, which was maintained for a duration of 5 days. Serum levels of PM20D1, interleukin (IL)-6, IL-1β, and C-reactive protein were measured on the sixth day after surgery using an enzyme-linked immunosorbent assay. Data on demographics, clinical statistics, lipid metabolism, nutritional parameters, and the prognostic nutritional index (PNI) were collected. Patients were followed up for 12 months, and both overall survival and disease-free survival were recorded.

**Results:**

In the low PNI group, the serum levels of PM20D1, albumin (ALB), and blood lymphocytes (BL) showed significant reductions. Pearson analysis revealed a negative correlation between PM20D1 and IL-6 levels, whereas a positive correlation emerged between PM20D1 and ALB and BL levels. Furthermore, PM20D1 demonstrated potential as a biomarker for diagnosing poor nutritional status (PNI < 43) in GC patients and was a risk factor for poor nutritional status in GC patients.

**Conclusion:**

Serum PM20D1 remarkably declined in GC patients after early EN and was associated with poor nutritional status.

## 1 Introduction

According to the Global Cancer Statistics for 2020, gastric cancer (GC) continues to represent a substantial global health concern, with an estimated one million new cases anticipated in 2020. It is projected that GC will account for 769,000 deaths, and the incidence rate is on the rise [1,2]. Surgical intervention constitutes the primary modality in the management of GC [3]. For patients undergoing this procedure, the tumor-induced physiological stress before surgery can lead to a diminished appetite, inadequate nutritional intake, and a hypermetabolic state associated with the tumor [4,5]. Furthermore, surgical intervention can exacerbate these effects by causing the depletion of nutritional proteins and a decline in immune function [6]. The resultant nutritional deficiencies and immune function impairment can heighten the susceptibility to infections and impede wound healing at the surgical site [7]. Nutritional disorders are a common issue in many malignancies and are often directly related to patient prognosis. Early nutritional support can help reduce the incidence of complications and improve overall treatment outcomes in cancer patients [8]. Additionally, immune disorders are often a consequence of tumor spread and progression, largely driven by the immunosuppressive environment created by tumors. This immunosuppression is facilitated by the increased production of inflammatory cytokines, which contribute to the persistence of a tumor-promoting environment [9]. Such mechanisms are common across various types of tumors and play a critical role in cancer progression and immune evasion. In addition to promoting tumor growth, increased production of inflammatory cytokines contributes to systemic effects such as cachexia, metabolic disorders, and other significant changes during disease progression. These cytokines create a pro-inflammatory and immunosuppressive environment that not only supports tumor progression but also impacts the overall health and metabolic state of cancer patients, often leading to a decline in quality of life and poorer prognosis [10]. Consequently, it is of paramount significance to initiate early postoperative nutritional support and continually evaluate the individual nutritional status and immune function of the patients.

Peptidase M20 domain-containing 1 (PM20D1) is a recently identified secretory enzyme that is secreted by adipocytes and notably enriched with uncoupling protein-1 [11]. PM20D1 has been found to enhance respiration in mice, leading to improved glucose homeostasis and increased energy expenditure [11]. Additionally, it has been observed that inhibiting miR-324-5p can elevate PM20D1 levels, consequently enhancing fat utilization and reducing weight in mice [12]. These studies underscore a strong connection between PM20D1 and energy expenditure, as well as lipid metabolism *in vivo*.

Furthermore, recent studies have shown the potential role of PM20D1 in modulating inflammatory responses. Dysregulated lipid metabolism and chronic inflammation are known contributors to cancer progression, including GC. GC is characterized by a complex interaction between tumor metabolism, inflammation, and nutritional status, which significantly affects patient prognosis [13,14]. PM20D1 may serve as a bridge linking metabolic dysregulation and inflammatory responses, making it a relevant target for investigation in GC patients. Understanding the relationship between serum PM20D1 levels, nutritional status, and inflammatory markers could provide insights into its potential as a biomarker for both disease progression and response to nutritional interventions. Moreover, reduced PM20D1 levels have been linked to increased secretion of inflammatory factors, compromised lipid metabolism, and adverse prognostic outcomes in conditions like atherosclerosis and gestational diabetes [15,16]. Nevertheless, there is currently a dearth of clinical studies investigating the interplay between PM20D1 in GC patients following enteral nutrition (EN), as well as its potential impact on patient prognosis.

### 1.1 Objectives

In this prospective observational study, our objective was to investigate the serum levels of PM20D1 in GC patients following early EN and its potential correlations with lipid metabolism (including total cholesterol [TC], triglycerides [TG], high-density lipoprotein cholesterol [HDLC], and low-density lipoprotein cholesterol [LDLC]), nutritional markers (albumin [ALB] and prealbumin [PA]), and inflammatory factors (including IL-6, IL-1β, and C-reactive protein [CRP]). The findings of this research may shed light on the clinical relevance of PM20D1 in GC patients undergoing EN and could offer fresh avenues for future research in GC treatment.

## 2 Methods

### 2.1 Subjects and study design

This prospective observational study included 180 GC patients who sought treatment at the Third Affiliated Hospital of Soochow University between May 2020 and July 2022. All patients underwent preoperative endoscopic observations, biopsy sampling, and histopathological examinations to confirm their GC diagnosis. This study was observational in nature, with no interventions imposed on patient treatment protocols. All treatments, including nutritional support, were administered in accordance with established clinical guidelines and tailored to individual patient conditions by the treating physicians. To ensure data consistency, only patients receiving similar nutritional support regimens were included, reflecting standard clinical practice without altering treatment decisions.

Patients were included if they received early EN via a nasoenteral tube placed during surgery, according to hospital guidelines. This intervention, administered at a rate of 50 mL/h for 5 days postoperatively, was part of routine care for patients with stable hemodynamics. This selection ensured consistency in nutritional support while maintaining adherence to individual clinical protocols.

The exclusion criteria were as follows: (1) patients who had received preoperative radiotherapy, chemotherapy, or immunotherapy; (2) patients classified as stage IV according to TNM criteria or aged over 70; (3) patients with congenital immunodeficiency, diabetes, or hyperthyroidism; (4) patients with severe infections, hepatic or renal dysfunction, or pulmonary disease before surgery; and (5) patients who discontinued EN therapy due to severe nutritional complications such as reflux or aspiration after surgery. All patients underwent GC resection following the latest clinical guidelines for GC diagnosis and treatment as per the Chinese Society of Clinical Oncology [17]. Postoperatively, no residual tumors or metastatic lesions were identified. This study has received approval from the Ethics Committees of The Third Affiliated Hospital of Soochow University (No.20200513), and all participants provided their informed consent to partake in this study.

For sample size calculation, we used the formula of *n* = *Z* ^2^ × *P*(1 – *P*)/*d* ^2^ to calculate the sample size. In this formula, *Z* represents the *Z*-score corresponding to the desired confidence level (*Z* = 1.96 for a 95% confidence level), *P* is the estimated proportion of the population with the characteristic of interest (*P* = 0.35 based on prior experience), and *d* is the allowable margin of error (*d* = 0.07 chosen according to our study requirements). Thus, the minimum sample size is *n* = *Z* ^2^ × *P*(1 – *P*)/*d* ^2^ ≈ 178. Considering that approximately 20% of patients might withdraw from the study, we aimed to enroll a total of 223 patients.

### 2.2 Early EN

As part of the observational study, no interventions were imposed on clinical treatment decisions. Nutritional support regimens were determined by the treating physicians based on standard hospital protocols and individual patient conditions. Prior to surgery, a gastric tube was inserted for decompression and monitoring. During the surgery, an enteral feeding tube was positioned through the jejunum. Specifically, after performing the anastomosis, the feeding tube was placed 20 cm below the Treitz ligament to facilitate EN postoperatively. This ensured that the EN could be safely and effectively administered starting from the first postoperative day. Commencing from the first-day post-surgery, EN support therapy was administered to patients via a nasoenteral tube placed during GC surgery. On the initial postoperative day, glucose saline was employed to maintain the patency of the EN tube. If the patient exhibited stable hemodynamics, an essential EN formula was administered to all patients starting on the first postoperative day at a rate of 50 mL/h, continuing for 5 days. The selection of the formula was based on clinical practice at our institution and was not a variable under evaluation in this study. Additionally, all patients received a daily infusion of 20 g of ALB within 48 h postoperatively. This approach ensured consistency across all patients, which was essential for evaluating the relationship between serum PM20D1 levels and nutritional status without the confounding effects of varying nutritional regimens. While immune-enhanced formulations are recommended in some contexts, our study focused on the effects of a consistent, widely used nutritional intervention.

Throughout the administration of the nutrition solution, patients were closely monitored for any adverse reactions related to nutritional support. In the event of symptoms such as bloating, abdominal pain, or discomfort during nutrition solution administration, or the development of post-administration diarrhea, the administration rate was adjusted, or the temperature and osmolarity of the nutrition solution were modified to alleviate the symptoms. The complete duration of EN support extended to 5 days, during which prophylactic anti-infective therapy was sustained. For patients with advanced-stage disease or high-risk features, adjuvant chemotherapy post-surgery was administered, typically using regimens like capecitabine combined with oxaliplatin or 5-fluorouracil. In cases with locally advanced tumors or close/positive surgical margins, postoperative radiotherapy was also included. Follow-up and monitoring occurred every 3–6 months, involving routine imaging (e.g., CT scans) and blood tests (e.g., tumor markers such as CEA or CA 19-9) to detect disease recurrence, with a follow-up duration of 12 months.

### 2.3 Blood sample measurement

PM20D1, interleukin (IL)-6, IL-1β, and CRP levels were assessed on the sixth day following surgery through enzyme-linked immunosorbent assay (ELISA). Within 24 h of admission, fasting cubital venous blood samples (5 mL) were collected from all participants. Blood samples were collected on the sixth postoperative day to measure serum PM20D1 levels, along with other relevant biomarkers. The ELISA kits used in this study were commercially available from MyBioSource and Abcam.

### 2.4 Observed indicators

A routine whole blood test was conducted using an automated biochemical analyzer (Hitachi 7600, Hitachi Corporation, Japan). The levels of TC, TG, HDLC, LDLC, serum ALB, blood lymphocyte (BL), and PA were measured and recorded. The prognostic nutritional index (PNI) [18,19] was calculated based on serum ALB and peripheral BL count, using the formula: PNI = (serum ALB, g/L) + (5 × BL count, 10^9^/L). Based on the PNI, all GC patients receiving EN were divided into two groups: the low PNI group (<43) and the high PNI group (≥43). Patients were followed up regularly at 1, 3, 6, and 12 months post-surgery. Each follow-up included clinical assessments, blood tests for relevant biomarkers, and imaging studies to monitor for disease recurrence, and the overall survival (OS) and disease-free survival (DFS) were recorded.

### 2.5 Statistical analysis

Mann–Whitney test (non-normal distribution data) or Student’s *t*-test (normal distribution data) was used for comparison between the two groups. The chi-square test was used for rates. Pearson’s rank correlation was used for correlation analysis. The role of serum PM20D1 in the prediction of patient prognosis was analyzed using ROC curve analysis. Kaplan–Meier (K–M) curve was used for analyzing the survival time. Logistic regression was performed for risk factors of poor prognosis. *P* < 0.05 regarded a significant difference. All data used SPSS 26.0 for statistical analyses.


**Informed consent:** All participants provided their informed consent to partake in this study.
**Ethical approval:** This study has received approval from the Ethics Committees of The Third Affiliated Hospital of Soochow University (No. 20200513).

## 3 Results

### 3.1 Clinical characteristics of all participants

**Figure 1:**
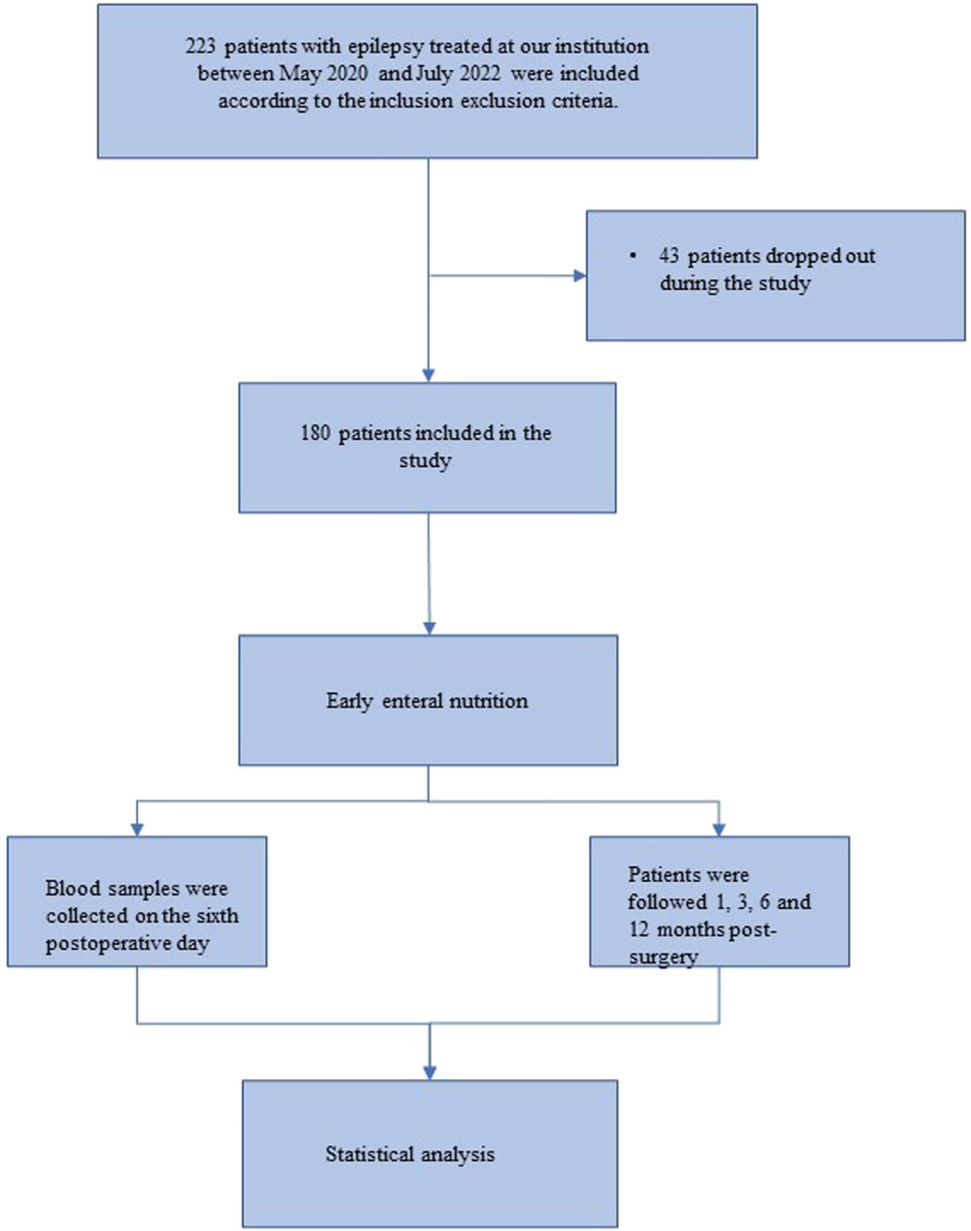
The flow diagram of the research.

The flow diagram is shown in Figure 1, this prospective observational study included 180 patients with GC who underwent postoperative early EN at our hospital. A total of 43 patients were excluded due to various reasons, including postoperative complications (*n* = 15), intolerance to EN (*n* = 12), and voluntary withdrawal from the study (*n* = 16). According to the PNI, all GC patients receiving EN were divided into the low PNI (<43) group (*n* = 52) and the high PNI (≥43) group (*n* = 128). When comparing demographic data and clinical data, we found no significant difference in age, sex, BMI, SBP, DBP, and lipid metabolism factors between the two groups (Table 1).

**Table 1:** Demographic and clinical data of all subjects

Variable	Low PNI group, *n* = 52	High PNI group, *n* = 128	*P*
Age (year)	54.5 (36–66)	54 (35–67)	0.940
Sex, female (%)	15 (48.4)	29 (45.3)	0.777
BMI	25.78 (21.24–29.00)	24.67 (21.02–29.29)	0.119
TNM stage			0.703
I-II, *n* (%)	27 (51.9)	63 (49.2)	
III, *n* (%)	25 (48.1)	65 (50.8)	
Type of surgery			0.494
Opened surgery, *n* (%)	24 (46.2)	53 (41.4)	
Endoscopic surgery, *n* (%)	28 (53.8)	75 (58.6)	
SBP (mmHg)	122.91 ± 13.08	122.30 ± 13.08	0.775
DBP (mmHg)	77.09 ± 8.88	79.45 ± 7.48	0.071
TC (mmol/L)	3.53 ± 0.55	3.56 ± 0.55	0.760
TG (mmol/L)	1.37 ± 0.14	1.33 ± 0.16	0.131
HDLC (mmol/L)	1.13 (0.97–1.24)	1.13 (0.93–1.25)	0.776
LDLC (mmol/L)	2.90 ± 0.35	2.84 ± 0.40	0.301

BMI – body mass index; SBP – systolic blood pressure; DBP – diastolic blood pressure; TC – total cholesterol; TG – triglyceride; HDLC – high-density lipoprotein cholesterol; LDLC – low-density lipoprotein cholesterol. Continuous data presented non-normal distribution were expressed by median (minimum to maximum) and analyzed by Mann–Whitney *U* test. Continuous data presented normal distribution were expressed by mean ± standard deviation and analyzed by Student’s *t*-test. The chi-square test was used for rates.

### 3.2 Serum levels of PM20D1 and nutritional-related indicators in GC patients

**Figure 2:**
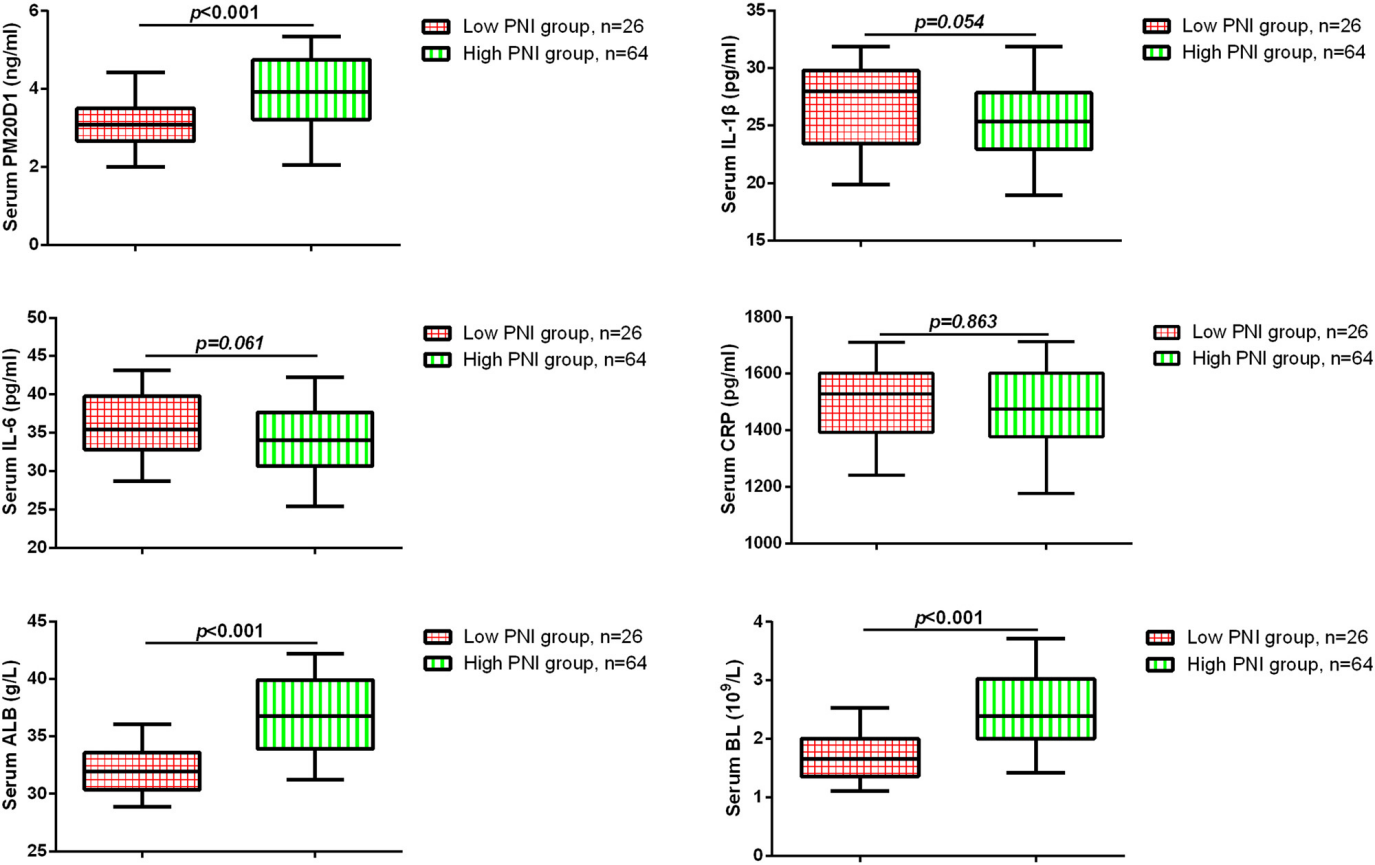
Comparisons of PM20D1 and other serum biomarkers in all subjects.

To further investigate the relationship between PM20D1 and nutritional status in GC patients, we measured the serum levels of PM20D1, IL-6, IL-1β, CRP, ALB, and PA. These markers allowed us to assess the inflammatory state (IL-6, IL-1β, CRP) and nutritional condition (ALB, PA) of the patients, providing a comprehensive understanding of their health status. As shown in Figure 2, compared to the high PNI group, the levels of PM20D1 in the serum of GC patients were significantly decreased in the low PNI group (*P* < 0.05). Additionally, the serum levels of ALB and BL were remarkably declined in the low PNI group compared to the high PNI group (*P* < 0.05). Pearson analysis supported a negative correlation between PM20D1 and the levels of IL-6, while a positive correlation between PM20D1 and the levels of ALB and BL (Table 2).

**Table 2:** Correlation analysis between PM20D1 and nutritional-related indicators

Variable	PM20D1	
	Pearson’s correlation	*P*
IL-1β (pg/mL)	−0.113	0.132
IL-6 (pg/mL)	−0.328	<0.001
CRP (pg/mL)	0.045	0.549
ALB (g/L)	0.287	<0.001
BL (10^9^/L)	0.368	<0.001
PA (mg/L)	−0.044	0.553

PM20D1 – peptidase M20 domain containing 1; IL – interleukin; CRP – C-reactive protein; ALB-albumin; BL – blood lymphocyte; PA – prealbumin. Pearson’s rank correlation was used for correlation analysis.

### 3.3 Predictive value of PM20D1 for the nutritional status and prognosis in GC patients

**Figure 3:**
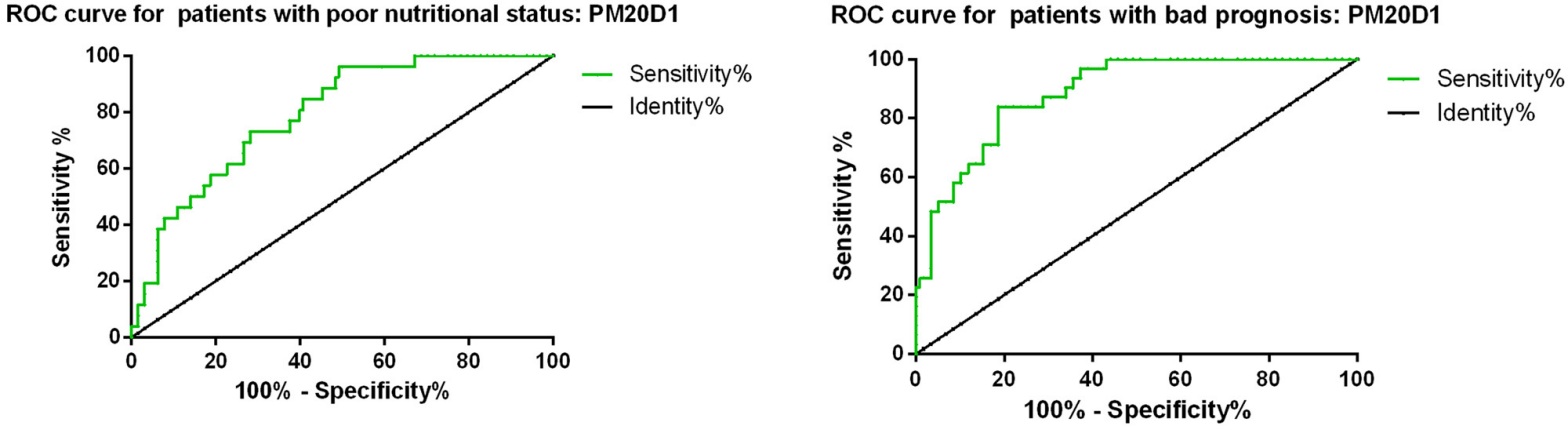
ROC curves for PM20D1 for the nutritional status and prognosis in GC patients.

We plotted the ROC curve to evaluate the diagnostic value of PM20D1 for distinguishing GC patients with poor nutritional status (PNI < 43). The results showed that the AUC for PM20D1 in diagnosing poor nutritional status in GC patients was 0.792, with a cutoff value of 3.28 nanograms/milliliter. The sensitivity was 73.4%, and the specificity was 69.2% (Figure 3). In addition, we found that PM20D1 could be a potential biomarker for predicting the prognosis of GC patients, the AUC of PM20D1 in predicting poor prognosis in GC patients was 0.886, with a cutoff value of 3.33 ng/mL, sensitivity of 81.4%, and specificity of 83.9%.

### 3.4 K–M curve analysis for serum PM20D1 and OS and DFS of GC patients

**Figure 4:**
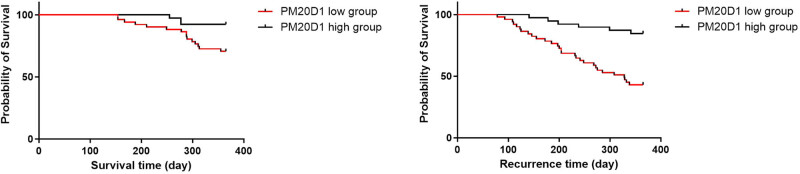
K–M curves for 12 months survival and recurrence time for GC patients with high/low serum levels of PM20D1.

The 12 months’ survival and relapse rates were also analyzed (average PM20D1 value, 3.69 ng/mL) by the K–M curve. The results showed that the low levels group had a shorter 1-year survival time and higher recurrence rate (Figure 4).

### 3.5 Risk factors of GC patients with poor nutritional status

Finally, we used the entry method for logistic regression to analyze the risk factors of GC patients with poor nutritional status. The results showed that age and PM20D1 were the independent risk factors for poor nutritional status in GC patients (Table 3).

**Table 3:** Logistic regression for risk factors of GC patients with poor nutritional status

Variables	Wald	Odds ratio	95% CI	*P*
Age	3.125	0.980	0.850–0.992	0.031
Sex	2.994	0.544	0.273–1.084	0.084
BMI	1.629	0.911	0.789–1.051	0.202
TNM stage	0.005	1.024	0.511–2.053	0.946
Tumor location	0.993	1.435	0.689–2.988	0.334
Surgical methods	0.056	0.919	0.458–1.845	0.812
SBP (mmHg)	0.001	0.999	0.942–1.018	0.989
DBP (mmHg)	3.814	1.044	0.995–1.109	0.051
TC (mmol/L)	0.096	0.867	0.353–2.131	0.756
TG (mmol/L)	0.091	1.637	0.067–39.987	0.762
HDLC (mmol/L)	0.420	6.810	0.021–2248.531	0.517
LDLC (mmol/L)	0.596	0.636	0.201–2.008	0.440
PA (mg/L)	0.367	1.005	0.988–1.023	0.545
PM20D1 (ng/mL)	24.171	8.439	3.606–19.750	<0.001
IL-1β (pg/mL)	0.078	0.979	0.846–1.133	0.780
IL-6 (pg/mL)	1.100	0.947	0.856–1.048	0.294
CRP (pg/mL)	0.633	0.988	0.994–1.002	0.426

BMI – body mass index; SBP – systolic blood pressure; DBP – diastolic blood pressure; TC – total cholesterol; TG – triglyceride; HDLC – high-density lipoprotein cholesterol; LDLC – low-density lipoprotein cholesterol; PM20D1 – Peptidase M20 domain containing 1; IL – interleukin; CRP – C-reactive protein; ALB – albumin; BL – blood lymphocyte; PA – prealbumin.

## 4 Discussion

Malnutrition affects 30–50% of patients hospitalized for medical and surgical reasons [20]. It has been reported that EN can decrease the incidence of complications and the duration of hospital stays in patients undergoing gastrointestinal cancer surgery [21]. Nevertheless, considering the high mortality rate associated with GC, additional serum markers are still necessary to evaluate nutritional status and forecast patient prognosis. Our study has revealed that serum PM20D1 can serve as a diagnostic tool for identifying poor nutritional status and predicting the prognosis of GC patients who undergo EN following surgery.

In recent years, an escalating number of studies have honed in on the significance of serum markers in patients undergoing early EN after surgery. Meta-analyses conducted by Yang and colleagues have demonstrated the enhanced effectiveness of early EN in elevating serum ALB and PA levels, expediting the recovery of gastrointestinal function, and reducing postoperative hospitalization duration in colorectal cancer patients [22]. Li et al. have substantiated the potential of early EN to diminish serum calcitonin gene-related peptide levels and the expression of the inflammatory cytokine IL-6, mitigate intestinal mucosal permeability, and enhance the prognosis of patients with severe acute pancreatitis [23]. Moreover, in the context of GC patients, Izumi and colleagues have observed that EN contributes to improved serum PA levels, and subsequent research has underscored the utility of elevated serum PA as an indicator for nutritional assessment and prognostic prediction [24]. Chen et al. have suggested that in GC patients receiving early EN, both immune and nutritional indices exhibit a significant increase on postoperative day 7, accompanied by a notable decrease in the expression of inflammatory cytokines [25]. In our study, we found that patients in the low PNI group had poorer nutritional status, which is consistent with the nutritional disorders observed in other types of tumors. This further emphasizes the importance of nutritional support in cancer treatment. However, no prior investigations have delved into the potential role of nutritional indices and serum cytokine levels in forecasting the prognosis of GC patients who receive EN. In our study, we have detected a noteworthy reduction in the levels of PM20D1, ALB, and BL in the serum of GC patients in the low PNI group. LDH is a well-established marker of metabolic disturbance and has been widely used in the assessment of various tumors, reflecting the metabolic state and prognosis of cancer patients. Elevated LDH levels are commonly observed in many cancer patients and are closely associated with metabolic disturbances [26]. In our study, we also observed changes in some nutritional indicators, which align with the metabolic disturbances indicated by LDH.

Targeting PM20D1 can inhibit lipid metabolism and promote lipid deposition [27]. Liu et al. also demonstrated that miR-324-5p promoted intramuscular lipid deposition through PM20D1 [28]. By upregulating PM20D1 expression, the PI3K/Akt signaling pathway can be activated, improving adipocyte breakdown and correcting metabolic abnormalities [29]. Yang et al. suggested that increased serum levels of PM20D1 were correlated with obesity-related glucose dysregulation, insulin resistance, and metabolic syndrome, making it a potential clinical biomarker for the diagnosis and monitoring of these diseases [30]. Furthermore, Hou et al. showed that decreased serum PM20D1 is associated with inflammation in patients with diabetes, indicating a link between PM20D1 and inflammatory processes [16]. These research findings suggest a close association between PM20D1 and lipid metabolism. Therefore, in our study, we measured serum levels of PM20D1, lipid metabolism factors, and inflammatory factors in all patients. The results showed a negative correlation between serum PM20D1 levels and IL-6. The correlation between PM20D1 and inflammatory markers such as IL-6 and CRP suggests a role for inflammation in modulating nutritional status in GC patients. Furthermore, it is well established that other cytokines also play critical roles in altering cellular metabolism, contributing to inflammation and metabolic disturbances as a consequence of tumor presence [31,32]. These cytokines collectively contribute to the metabolic changes observed in cancer patients, thereby exacerbating disease progression and impacting overall prognosis. The observed association between PM20D1 and inflammation-related markers highlights its potential role in tumor biology. Additionally, other enzymes, such as matrix metalloproteinases, have been shown to play important roles in tumor progression, primarily through their involvement in extracellular matrix remodeling and promoting metastasis [33].

Additionally, we found a positive correlation between serum PM20D1 levels and ALB levels, indicating a relationship between PM20D1 and nutritional indicators. Likewise, a study by Noronha et al. investigating the modulation of DNA methylation patterns by zinc (Zn) also suggests that the lower methylation of the PM20D1 gene might indicate an interplay between DNA methylation and nutritional status, providing a new perspective for understanding the genetic mechanisms of nutrition [34]. Nonetheless, there is a paucity of research centering on the role of PM20D1 in determining patient nutritional status and its predictive capacity about the prognosis of GC patients. In our study, in the K–M survival analysis, the cohorts were stratified directly based on serum PM20D1 levels, independent of the PNI. The observed differences in 12-month survival and relapse rates between high and low PM20D1 groups indicate that PM20D1 serves as an independent prognostic marker. While PNI is an established metric of nutritional status, our analysis highlights the potential of PM20D1 to provide additional prognostic insights, specifically related to metabolic regulation and inflammation in GC patients. Additionally, we have discerned that serum PM20D1 holds potential as a prognostic indicator for GC patients who have undergone early EN following surgery. Our analysis aimed to evaluate the prognostic significance of PM20D1 in GC patients receiving EN, as its role in metabolic regulation and inflammatory response may have direct implications for survival. We acknowledge that including additional clinical variables could offer further insight, but our study focused specifically on the potential of PM20D1 as an independent prognostic factor.

Our findings suggested that serum PM20D1 levels correlated with nutritional status and inflammatory markers in GC patients undergoing early EN. Compared to other inflammatory biomarkers such as CRP and IL-6, which are well-established markers of systemic inflammation, PM20D1 appears to offer a unique perspective by linking metabolic processes with inflammation. CRP and IL-6 have been widely studied in the context of cancer-related inflammation, but PM20D1, as a lipid metabolism-related enzyme, may provide additional information on metabolic dysregulation in cancer patients. Adiponectin, another biomarker involved in metabolic regulation, has also been linked to cancer progression [35]. However, while adiponectin levels generally decrease in cancer patients, PM20D1 levels reflect a more specific interplay between lipid metabolism and immune response. This comparison suggests that PM20D1 might serve as a complementary biomarker, providing information that is not captured by traditional inflammatory markers alone. Future studies should focus on evaluating PM20D1 alongside these biomarkers to determine its potential as an independent or additive predictor of clinical outcomes.

In our study, we propose that serum PM20D1 could serve as a diagnostic indicator for evaluating nutritional status in GC patients receiving EN. Compared to traditional nutritional risk tools such as NRS 2002, PM20D1 offers a biomarker-based approach that may provide insights into the patient’s metabolic and inflammatory state, which are critical factors in recovery and prognosis but may not be fully captured by clinical assessments. Traditional tools like NRS 2002, on the other hand, offer a broader assessment based on patient history, physical examination, and clinical factors, providing a more holistic view of nutritional risk. One advantage of PM20D1 is its potential to detect subtle metabolic disturbances related to lipid metabolism and inflammatory responses, which could serve as early indicators of poor nutritional status. However, its limitations lie in the fact that PM20D1 is not yet a standard measure in clinical practice, and its prognostic value requires further validation through larger-scale studies. Additionally, while NRS 2002 is widely used and validated in clinical settings, PM20D1 could act as a complementary biomarker to enhance nutritional risk stratification, particularly in patients undergoing cancer treatment.

## 5 Limitations

The present study has several limitations. First, the modest sample size may limit the generalizability of our findings. Secondly, we focused on a limited set of inflammatory factors, potentially overlooking other relevant variables. While our study examined serum PM20D1 levels and postoperative nutritional status, we acknowledge the importance of preoperative nutritional risk assessments, such as the NRS 2002 score, which was not included in our data collection. Additionally, PM20D1 assays are not widely available in clinical practice, and their cost may limit broader use. Due to the short follow-up period, our OS results may not fully reflect long-term outcomes. Future studies with extended follow-up are necessary to provide more comprehensive survival data. While our findings suggest that PM20D1 is a promising biomarker for GC, its specific role and regulatory mechanisms within this disease context require further elucidation. To establish PM20D1 as a GC-specific marker, future studies could employ in-depth gene expression analyses across different cancer and non-cancer tissues to determine the specificity of PM20D1 in GC. In addition, *in silico* modeling could be used to identify potential cancer-specific regulatory pathways and protein interactions involving PM20D1. These approaches would provide a clearer understanding of its unique function in GC and help establish its role as a disease-specific biomarker.

## 6 Conclusion

Our findings indicated that PM20D1 levels were remarkably declined in GC patients after early EN with poor nutritional status. In addition, serum levels of PM20D1 could be a potential predictive biomarker of the prognosis in GC patients after early EN. This study might provide new targets and a comprehensive approach to treatment in GC patients.

## Abbreviations

ALB: albumin
BL: blood lymphocyte
BMI: body mass index
CRP: C-reactive protein
CSCO: Chinese Society of Clinical Oncology
DBP: diastolic blood pressure
DFS: disease-free survival
ELISA: enzyme-linked immunosorbent assay
EN: enteral nutrition
GC: gastric cancer
HDLC: high-density lipoprotein cholesterol
IL: interleukin
K–M: Kaplan–Meier
LDLC: low-density lipoprotein cholesterol
OS: overall survival
PA: prealbumin
PM20D1: peptidase M20 domain containing 1
PNI: prognostic nutritional index
ROC: receiver operating characteristic
SBP: systolic blood pressure
TC: total cholesterol
TG: triglyceride
TNM: tumor, node, metastasis
UCP1: uncoupling protein-1
